# Lifestyle and risk of developing myopia in school children in Chongqing, China

**DOI:** 10.3389/fmed.2024.1439833

**Published:** 2024-10-08

**Authors:** Ruili Li, Jing Zhang, Yong Zhang, Wensheng Tang, Dan Ao, Li He, Kun Yang, Xiaoya Qi

**Affiliations:** ^1^Health Medicine Center, The Second Affiliated Hospital of Chongqing Medical University, Chongqing, China; ^2^School of Public Health, Chongqing Medical University, Chongqing, China

**Keywords:** myopia, cross-sectional study, lifestyle factors, schoolchildren, China

## Abstract

**Introduction:**

Myopia has emerged as a leading global reason for poor vision in children and adolescents. this study aims to investigate the influence of lifestyles on myopia in schoolchildren in Chongqing, China.

**Methods:**

This cross-sectional study was conducted on primary and junior high school graduates in Chongqing City, China. Students who came to the assigned hospital for the physical examination of their upgrade school enrollment were recruited. In addition to regular examination and eyeusing questionnaire, visual acuity and non-cycloplegic autorefraction were measured.

**Results:**

Of all 1806 eligible students, 1,623 students (89.87%) were included in the analysis. The prevalence of myopia in elementary and junior middle school graduates was 73.1 and 81.8%, respectively. According to the multivariate logistic regression analysis, 1 h more homework (OR = 1.272, *p* = 0.032), attending out-school courses (OR = 1.973, *p* = 0.006), frequently checking of eyes (OR = 1.337, *p* = 0.015) and using eye-protecting lamp (OR = 2.528, *p* < 0.001) were more likely to be associated with myopia (*p* < 0.05). While 1 h more outdoor activity in weekday (OR = 0.811, *p* = 0.033) and weekend (OR = 0.796, *p* = 0.034) were less likely to have myopia.

**Conclusion:**

The current prevalence of myopia among elementary and junior high school students in Chongqing is high. Academic pressures, bad habits of using eyes, and limited time for outdoor activity mainly contribute to the epidemic of myopia. Various policies in and out of schools related to reducing academic pressures, increasing outdoor activities, and improving eye habits may help control the prevalence of myopia in teenagers.

## Introduction

Myopia is a significant public health issue and has emerged as a leading global reason for poor vision in children and adolescents (1). The prevalence of myopia has markedly increased over the past half-century. The highest prevalence of myopia exists in East Asia, especially in China (2). Among people with myopia, children and adolescents are more prominent. The pooled prevalence of myopia in individuals aged 3 years to 19 years in the period from 1998 to 2016 was 37.7% (3).

Moreover, the prevalence of myopia has gone without sign of stopping in recent decades. The globe myopia progression was −0.44D (diopters) per year in children (4). Myopia prevalence in 2050 among children and adolescents aged 3–19 years in China was estimated to be higher than 80% (3).

Myopia undeniably increases the risk of several ocular complications, such as cataracts, glaucoma, retinal detachment, and macular degeneration (5). As far as children are concerned, myopia affects school performance, limits employability, and impairs an individual’s quality of life. Socioeconomically, due to related ocular health issues and vision impairment, the costs of myopia are also significant. The potential global productivity loss associated with the burden of visual impairment in 2015 was estimated at US$244 billion from uncorrected myopia (6). Due to those reasons, on July 23, 2021, the United Nations General Assembly approved a Vision for Everyone resolution, calling on its members “to make eye care an integral part of universal health coverage” (7). For children and adolescents, the prevention and control of myopia are crucial because of the profound impact of children’s myopia on eye health care in an individual’s whole life.

Myopia is believed to be the result of genetic and environmental factors, and environmental factors play a vital role in the onset of myopia in children and adolescents (1). Because genetic factors are immutable, environmental factors relate to personal lifestyle and are variable, and environmental and lifestyle factors are more important than genetic factors in myopia control. According to previous studies, there are various environmental factors related to myopia in children and adolescents. However, the conclusions on some environmental factors related to myopia could be more consistent, such as the effect of near work on myopia (8–14). Those inconsistencies about myopia or protective factors may be due to different living contexts in previous studies. Therefore, more studies are needed to explore or confirm various factors related to myopia, especially in different settings and populations. The present study aimed to assess the factors of myopia related to lifestyle for children and adolescents in Chongqing, a southwestern city in China. It can provide valuable evidence for the effective prevention and accurate management of myopia in children and adolescents.

## Methods

### Study participants

In the summer of 2023, a cross-sectional survey was conducted on primary and junior high school graduates who visited the hospital for their enrollment physical examination. Ethical approval (No. CAF52704054B) for this study was obtained from the Ethics Committee of Chongqing Medical University, according to the principles of the Declaration of Helsinki. Written consent was obtained from all subjects involved (the participants’ legal guardians/next of kin).

Students who refused participation or had a medical history of eye diseases such as strabismus, amblyopia, high astigmatism, ocular inflammation, ocular trauma, corneal disease, congenital cataract, choroid, or retinal disorders were excluded.

### Ocular examinations

Ocular examinations contained visual acuity, slit-lamp examination, direct ophthalmoscopy, and noncycloplegic refraction. All subjects underwent measurement of uncorrected distance visual acuity (UCDVA) at 5 m (standard logarithmic visual acuity E chart) and recorded in Log MAR scores. An auto refractometer (HRK-7000A, Huvitz Co., Ltd.) was used to measure noncycloplegic refraction in a darkened room. Each eye of each student was measured at least thrice. If the difference of refractive error (RE) between measurements reached 0.50 diopters (D) or above, additional examinations were conducted. The mean measurement values were used for analysis.

### Physical examination

Physicians from the Department of Health Medicine Center conducted other conventional physical examinations, including height (centimeters), weight (kilograms), and blood pressure (millimeter mercury column). Body mass index (BMI) was calculated by dividing weight by height (kg/m2).

### Questionnaire survey

Demographic, sociological, and behavioral information about eyesight was collected through a self-administered web-based questionnaire at the site. The same investigating team guided all survey questionnaires to reduce individual errors.

### Definitions and classifications

Visual acuity<0 LogMAR was considered normal vision. The spherical equivalent refraction (SER) was converted by adding the spherical refraction and half the cylindrical refraction. Myopia was defined as UCVA >0 logMAR in the right eye with SER ≤ −0.50 D (15). Because the right eye and left eye are highly correlated, the right eye was used in the analysis if necessary (16).

Overweight and obesity were defined according to the national comprehensive evaluation standards of children and adolescents’ development. For primary school graduates at age 12, the BMI for overweight is 21.0–24.7 and 21.9–24.5 for males and females, respectively, and the BMI for obesity is > = 24.7 and >=24.5 for males and females, respectively. For junior middle school graduates at age 15, the BMI for overweight is 23.1–26.9 and 23.4–26.9 for males and females, respectively, and the BMI for obesity is > = 26.9 for both sexes (17).

### Quality control

Before the study started, members of the research team, including two experienced ophthalmologists, two qualified optometrists, and three postgraduates, were trained. All instruments were checked and adjusted before the examination.

### Data analysis

Categorical and continuous variables were summarized as proportions or mean ± standard deviation (SD) and compared between subjects with and without myopia using the chi-square test or the analysis of variance (ANOVA). The association between lifestyle factors and the occurrence of myopia was analyzed using multivariate logistic regression. All statistical analyses were conducted using the software SPSS 26.0. A two-tailed *p*-value below 0.05 was considered statistically significant.

## Results

### Prevalence of myopia

Of all 1806 eligible students, 183 were excluded due to missing data, and 1,623 students (89.87%) were included in the analysis: 871 elementary school graduates (ESGs) and 752 junior middle school graduates (JSGs). Overall, myopia rates in the right eye for the ESGs and JSGs in this study were 73.1 and 81.8% (*p* < 0.001), respectively. Furthermore, for the left eye, the myopia for ESGs and JSGs in this study was 66.6 and 77.0% (*p* < 0.001), respectively. The prevalence of myopia was significantly high in JSGs for both eyes.

### Characteristics of subjects

The characteristics of subjects were summarized and compared by eyesight status in Table 1. For gender, there was no difference in JSG, but females were more likely to have myopia in ESG (*p* < 0.05). Myopia was not different from regular eyesight counterparts in height, weight, BMI, SBP, and DBP. Furthermore, the parents’ education levels were not related to myopia. However, parents’ myopia and higher academic achievement (the prestige of the schools they were admitted to in the next grade) were more likely to be myopia in ESGs and/or JSGs (*p* < 0.05).

**Table 1:** Characteristics of school children by eyesight status (*N* = 1,623).

	ESG		*P*_1_	JSG		*P*_2_
	Normal (*N* = 234)	Myopia (*N* = 637)		Normal (*N* = 137)	Myopia (*N* = 615)	
Gender (male, *n*%)	137 (58.5)	314 (49.3)	**0.018**	75 (54.7)	312 (50.7)	0.450
Age (year)	12.05 ± 1.00	12.04 ± 0.81	0.838	14.99 ± 0.98	14.98 ± 0.86	0.866
Height (cm)	157.9 ± 7.6	158.9 ± 7.1	0.098	167.7 ± 7.7	167.1 ± 8.3	0.470
Weight (Kg)	49.3 ± 12.11	48.7 ± 10.6	0.408	59.5 ± 12.9	59.1 ± 13.2	0.757
BMI	19.64 ± 3.80	19.15 ± 3.35	0.058	21.06 ± 3.64	21.04 ± 3.93	0.963
SBP (mmHg)	109.8 ± 11.1	110.0 ± 11.4	0.780	115.5 ± 12.9	114.5 ± 11.8	0.395
DBP (mmHg)	65.2 ± 6.9	65.3 ± 7.5	0.927	67.4 ± 8.5	67.2 ± 8.0	0.853
Without sibling (yes)	102 (43.6)	285 (444.7)	0.818	63 (46.0)	280 (45.5)	0.925
Parents myopia			**<0.001**			**<0.001**
1, Father (*n*, %)	35 (15.0)	119 (18.7)		23 (16.8)	110 (17.9)	
2, Mother (*n*, %)	37 (15.8)	160 (25.1)		16 (11.7)	128 (20.8)	
3, Both (*n*, %)	33 (14.1)	141 (22.1)		17 (12.4)	123 (20.0)	
Mother education			0.783			0.434
1, Low (*n*, %)	98 (42.1)	285 (44.7)		82 (59.9)	333 (54.2)	
2, Middle (*n*, %)	124 (53.2)	320 (50.2)		50 (36.5)	261 (42.5)	
3, High (*n*, %)	11 (4.7)	32 (5.0)		5 (3.6)	20 (3.3)	
Father education			0.248			0.623
1, Low (*n*, %)	112 (48.1)	268 (42.1)		75 (54.7)	322 (52.4)	
2, Middle (*n*, %)	107 (45.9)	319 (50.1)		56 (40.9)	252 (41.0)	
3, High (*n*, %)	14 (6.0)	50 (7.8)		6 (4.4)	40 (6.5)	
Academic achievement			**0.054**			**0.002**
1, Low (*n*, %)	21 (9.0)	42 (6.6)		44 (32.1)	115 (18.7)	
2, Middle (*n*, %)	156 (64.1)	371 (58.2)		54 (39.4)	289 (47.0)	
3, High (*n*, %)	63 (26.9)	224 (35.2)		39 (28.5)	211 (34.3)	

ESG, elementary school graduates; JSG, junior middle School Graduates, P1 for ESG groups comparing, P2 for JSG groups comparing; Myopia: UCVA > 0 logMAR & SER < = − 0.5 in the right eye. Bold values means *P* < 0.05.

### Lifestyle factors and myopia

Lifestyle factors were presented in Table 2 by their eyesight categories. In those factors related to learning, homework (time spent in hours) was more in myopia, especially in JSGs (*p* = 0.002), breaks in eye-using were less taken in myopia, especially in JSG, attending out-school courses was more often in JSG myopia (*p* = 0.003). Less outdoor activity on weekdays or weekends was found in myopia in ESGs and/or JSGs for those factors about physical activities. The weekday outdoor activity hours were significantly less in JSGs (*p* = 0.005), while weekend outdoor activity hours were significantly less in ESGs (*p* = 0.016). It was unexpected that some eyesight-protection measures, such as eyesight-protection lamps or routine eyesight-checking, were more common in myopia students, especially the eyesight-protection lamp used for ESGs (*p* < 0.001). Other factors, such as sleeping time, screen time, and lodging in school, were not different between normal and abnormal eyesight groups in ESGs and JSGs.

**Table 2:** lifestyle factors of school children by eyesight status (*N* = 1,623).

Variable	ESG		*P*_1_	JSG		*P*_2_
	Normal (*N* = 234)	Myopia (*N* = 637)		Normal (*N* = 137)	Myopia (*N* = 615)	
Homework in hours			0.590			**0.002**
1, <1 h	50 (21.4)	125 (19.6)		19 (13.9)	34 (5.5)	
2, 1 ~ 2 h	117 (50.0)	300 (47.1)		38 (27.7)	148 (24.1)	
3, 2 ~ 3 h	49 (20.9)	161 (25.3)		39 (28.5)	189 (30.7)	
4, > = 3 h	18 (7.7)	51 (8.0)		41 (29.9)	244 (39.7)	
Breaks in using eyes			0.2237			**0.013**
1, Seldom	45 (19.3)	137 (21.5)		22 (16.1)	174 (28.3)	
2, sometime	137 (58.8)	392 (61.5)		96 (70.1)	366 (59.5)	
3, Often	51 (21.9)	108 (17.0)		19 (13.9)	75 (12.2)	
Attending out-school course (yes)	209 (89.3)	556 (87.3)	0.483	102 (74.5)	525 (85.4)	**0.003**
Outdoor activity (weekday)			0.174			**0.005**
1, <1 h	51 (21.8)	186 (29.2)		37 (27.0)	235 (38.2)	
2, 1 ~ 2 h	125 (53.4)	301 (47.3)		63 (46.0)	264 (42.9)	
3, 2 ~ 3 h	36 (15.4)	90 (14.2)		16 (11.7)	72 (11.7)	
4, > = 3 h	22 (9.4)	59 (9.3)		21 (15.3)	44 (7.2)	
Outdoor activity(weekend)			**0.016**			0.118
1, <1 h	28 (12.0)	134 (21.0)		34 (24.8)	195 (31.7)	
2, 1 ~ 2 h	103 (44.0)	269 (42.2)		55 (40.1)	259 (42.1)	
3, 2 ~ 3 h	57 (24.4)	137 (21.5)		22 (16.1)	85 (13.8)	
4, > = 3 h	46 (19.7)	97 (15.2)		26 (19.0)	76 (12.4)	
Sleeping time			0.319			0.828
1, <5 h	0 (0.0)	2 (0.3)		1 (0.7)	10 (1.6)	
2, 5 ~ 7 h	10 (4.3)	37 (5.8)		62 (45.3)	270 (43.9)	
3, 7 ~ 9 h	167 (71.4)	473 (74.3)		68 (49.6)	313 (50.9)	
4, > = 9 h	57 (24.4)	125 (19.6)		6 (4.4)	22 (3.6)	
Screen time(weekday)			0.495			0.138
1, <1 h	123 (52.6)	325 (51.1)		63 (46.0)	333 (54.2)	
2, 1 ~ 2 h	74 (31.6)	187 (29.4)		42 (30.7)	142 (23.1)	
3, 2 ~ 3 h	22 (9.4)	84 (13.2)		14 (10.2)	77 (12.5)	
4, > = 3 h	15 (6.4)	40 (6.3)		18 (13.1)	62 (10.1)	
Screen time(weekend)			0.155			0.111
1, <1 h	38 (16.9)	118 (19.0)		5 (4.4)	59 (9.4)	
2, 1 ~ 2 h	105 (44.6)	242 (39.0)		37 (26.3)	144 (24.0)	
3, 2 ~ 3 h	55 (23.6)	147 (22.5)		32 (24.1)	157 (25.1)	
4, > = 3 h	35 (14.9)	130 (19.6)		63 (45.3)	255 (41.4)	
Lodging in school(yes)	8 (3.4)	20 (3.1)	0.830	30 (21.9)	144 (23.4)	0.738
Eyesight-checking			0.142			0.640
0 time/year	12 (5.4)	25 (3.7)		7 (5.1)	20 (3.0)	
1 times/year	145 (62.2)	362 (56.3)		82 (60.6)	392 (62.6)	
2 times/year	63 (27.4)	185 (28.5)		41 (29.2)	168 (27.1)	
3 or more times/year	12 (5.0)	60 (11.5)		7 (5.1)	35 (7.3)	
Using eyesight-protection lamp(yes)	52 (22.2)	231 (36.3)	**<0.001**	39 (28.5)	187 (30.4)	0.682
Using eyesight-protection desk (yes)	67 (28.6)	199 (31.2)	0.371	25 (18.2)	133 (21.6)	0.494

ESG, elementary school graduates; JSG, junior middle school graduates, P_1_ for ESG groups comparing, P_2_ for JSG groups comparing. Bold values means *P* < 0.05.

### Multivariable analysis of lifestyle factors and myopia

Multiple logistic regression analysis showed that homework amount, attending the out-school courses, routing eyesight-checking, eye-protection lamp use, breaks in eye-using, and outdoor activities were associated with the myopia of ESG or JSG.

For ESG, 1-h outdoor activities increment on weekends decreased the risk of myopia by about 19% (OR: 0.811, 95% CI: 0.669–0.984), more time eyesight-checking related to more myopia (OR: 1.337, 95% CI: 1.058–1.69), eye-protection lamp using was found also positively related to myopia (OR: 2.528, 95% CI: 1.678–3.811).

For JSG, 1-h increment of homework increased 27% myopia (OR: 1.272, 95% CI: 1.021–1.586), attending out-school courses increased 97% risk of myopia (OR: 1.973, 95% CI: 1.221–3.188), and 1-h outdoor activities increment in weekday decreased the risk of myopia about 21% (OR: 0.796, 95% CI: 0.591–0.987) (Table 3).

**Table 3:** Multivariable logistic regression analysis of lifestyle factors of myopia.

	OR*	95%CI	*P*
ESG			
Homework in hours (1-h increment)	0.936	0.521–1.681	0.825
Attending out-school course (yes vs. no)	0.739	0.455–1.199	0.221
Breaks in eye-using (often vs. seldom)	0.869	0.670–1.126	0.288
Outdoor activities in weekday (1-h increment)	1.020	0.828–1.256	0.853
Outdoor activities in weekend (1-h increment)	**0.811**	0.669–0.984	**0.033**
Sleeping time (2-h increment)	0.786	0.566–1.090	0.149
Screen time in weekday (1-h increment)	1.033	0.846–1.262	0.747
Screen time in weekend (1-h increment)	1.142	0.947–1.376	0.166
Eyesight-checking (one-time increment)	**1.337**	1.058–1.690	**0.015**
Using eye-protection lamp (yes vs. no)	**2.528**	1.678–3.811	**<0.001**
Using eye-protection desk (yes vs. no)	0.833	0.562–1.235	0.363
Lodging in school (yes vs. no)	1.275	0.549–2.960	0.572
JSG			
Homework in hours (1-h increment)	**1.272**	1.021–1.586	**0.032**
Attending out-school course (yes vs. no)	**1.973**	1.221–3.188	**0.006**
Breaks in eye-using (often vs. seldom)	0.743	0.528–1.046	0.089
Outdoor activities in weekday (1-h increment)	**0.796**	0.591–0.978	**0.034**
Outdoor activities in weekend (1-h increment)	0.95	0.752–1.199	0.665
Sleeping time (2-h increment)	1.064	0.762–1.486	0.714
Screen time in weekday (1-h increment)	1.001	0.810–1.236	0.994
Screen time in weekend (1-h increment)	0.966	0.776–1.201	0.753
Eyesight-checking (one-time increment)	1.041	0.766–1.414	0.799
Using eye-protection lamp (yes vs. no)	1.016	0.623–1.659	0.948
Using eye-protection desk (yes vs. no)	1.138	0.663–1.956	0.639
Lodging in school (yes vs. no)	0.852	0.530–1.370	0.509

ESG, elementary school graduates; JSG, junior middle school graduates. *Adjusted by gender, parent’s myopia, parent’s education, academic achievement, sibling numbers, height, weight, SBP, DBP, BMI. Bold values means *P* < 0.05.

## Discussion

The current cross-sectional study’s results show that the incidence of myopia among primary and middle school students in Chongqing City was 73.1 and 81.8%, respectively, which was higher than previous studies (18, 19).

Except for some demographic, sociological, and genetic variables such as gender, parent’s myopia, and parent’s education level, lifestyle factors may also play essential roles in the development of myopia in students at different stages. In this study, we found that lifestyle factors such as the amount of homework, attending out-of-school courses, breaks in eye-using, outdoor activities, and eye-protection measures had relationships with the prevalence of myopia. With multivariable analysis, we further identified that 1-h outdoor activities increment on weekends (OR = 0.811, *p* = 0.033), regular eyesight-checking (OR = 1.337, *p* = 0.015), and eye-protection lamp using (OR = 2.582, *p* < 0.001) related to myopia in ESGs. In contrast, 1-h increment of homework (OR = 1.272, *p* = 0.032), attending the out-school course (OR = 1.973, *p* = 0.006), and 1-h increment of outdoor activities on weekdays (OR = 0.796, *p* = 0.034) were related to myopia in JSG.

The effect of near work on myopia has been controversial, with some studies failing to report a significant correlation between myopia and near work (8–10). In our study, homework, a kind of near work (short distance use of eyes), was significant for incident myopia in junior middle school students. Several previous studies supported our findings, by reporting that children who spent more time on reading or writing have a higher prevalence of myopia (11–14). Moreover, few breaks during reading and writing are also a risk factor for myopia (20, 21). Our study partly confirmed the findings of previous research. In our study, breaks in eye use were a protective factor for myopia in junior high school students. The after-school tutorial deserves special attention in education since it reinforces rote learning and an examination-oriented competitive culture within schooling (22). An epidemiological link between additional tutoring and the development of myopia has been established (23–26). Our study further confirmed this link between out-of-school courses and myopia in junior middle school students. The possible mechanism of heavy school work to myopia may be the excessive reading at a short distance without breaks to myopia, which can cause retinal images to remain in a defocused state for a long time, a condition called accommodative lag (27). However, the adjustment to the blurred image will lead to the increase of the accommodative lag, and the long-term accommodative lag induces the retina to produce some neurotransmitters or growth factors to regulate the inappropriate growth of the axial length of the eye, thus leading to the progression of myopia (21). Due to the fierce competition in school, extra homework and extracurricular courses and lack the awareness to relax and rest their eyes during study lead to the high prevalence of myopia among teenagers. Therefore, the current policy about reducing academic competition by banning extracurricular in China at the compulsory education stage while promoting more time outdoors activities may effectively reduce myopia in the coming years.

Our findings also showed that outdoor weekend activity was a protective factor for myopia in primary school students, and outdoor activity on weekdays was a protective factor for junior middle school students. Numerous studies on student populations consistently demonstrated that outdoor activity is a protective factor for school children’s myopia, more outdoor activity with less time near work was found to provide the most robust protection against myopia onset (28–30). even in countries with low prevalence of myopia. For example, Jones et al. (9) reported that myopia prevalence was 5.4% (CI 3.8–6.9%) in students with European Caucasian parents and 39.8% (CI 33.8–45.7%) in those with East Asian parents. Students of East Asian origin spent significantly more time on near–work and less time on outdoor leisure activity than Caucasian children.

It is worth noting that, in our study, screen time and sleep time were not associated with myopia. This may due to that entertainments devices with screen, such as smartphones, were strictly controlled by parents and information about screen time was not accurate. Similarly, two meta-analyses showed that the relationships between myopia and screen time have insufficient evidence (31, 32). To date, the association between insufficient sleep duration and the incidence of myopia also remains insufficient (33). Interestingly, some eye protection measures such as eye-protection lamps and desks or regular eyesight checking were found in this study to be positively related to myopia. This may be due to a reversal causality, while parents resorted to those eye-protection products or increased the eyesight checking frequency when their children had short eyesight problems. On the other hand, this may imply that eye-protection products have slight effectiveness in myopia control. Previous studies by Pan et al. found that compared with children who used incandescent or fluorescent lamps, children who used LED lamps for homework had a higher prevalence of myopia (34). In addition, most of the lamps claimed to protect the eyes in the Chinese market have varying degrees of stroboscopic, and such lamps may unconsciously damage the eyesight of children and teenagers (35).

There are some strengths for this study. First, the subject came from various schools with different academic performances, which may allow us to identify the possible factors of myopia. Second, the sample size is big enough to have enough power to test those factors on myopia. Third, we added the spherical equivalent refraction rather than the visual acuity chart test only, which was more accurate for the diagnosis of myopia. However, this study has some limitations. First, all subjects came from one hospital, which may not represent all teenagers in Chongqing; a multi-center study is warranted. Second, we used non-dilatating computer optometry for spherical equivalent refraction measurements due to time and workforce. However, cycloplegic automatic optometry is the preferred method for determining the degree of myopia. The prevalence of myopia in the current study may have been overestimated. Future studies using automated optometry with ciliary paralysis are needed. Third, because lifestyle information was collected through questionnaires, there may be a recall bias about long-term conditions, and, because this is a cross-sectional study, the causal links cannot be proved. Therefore, a cohort study may be needed to confirm our findings in the future.

## Conclusion

The prevalence of myopia among elementary and junior high school students in Chongqing is relatively high. Reducing academic pressures, increasing outdoor activities, and improving eye-using habits are the keys to curbing the prevalence of myopia in children.

## Data Availability

The raw data supporting the conclusions of this article will be made available by the authors, without undue reservation.
